# Assembly of resistant starch type 5 in two amylose matrices: effects of fatty acid chain length and matrix background on structural characteristics

**DOI:** 10.1016/j.fochx.2026.103942

**Published:** 2026-05-02

**Authors:** Mingyi Zhang, Caiming Li, Jihong Huang, Jingbo Zhou

**Affiliations:** a State Key Laboratory of Food Science and Resources, Jiangnan University, Wuxi, Jiangsu 214122, China; b Rice Research Institute, Guangdong Academy of Agricultural Sciences, Guangzhou 510642, China; c State Key Laboratory of Crop Stress Adaptation and Improvement College of Agriculture, Henan University, Kaifeng 475004, China

**Keywords:** Amylose–lipid complexes, Fatty acid chain length, Interactions, Fine structure

## Abstract

This study examined resistant starch type 5 (RS5) assembly in two amylose-rich maize matrices with comparable amylose contents (>91%) but distinct processing histories and apparent molecular states: autoclave-treated amylose matrix (AAM) and butanol-extracted amylose matrix (BAM). Each matrix was complexed with saturated fatty acids of different chain lengths (C6, C12, and C20). In both matrices, increasing fatty acid chain length was consistently associated with higher lipid content and V-type crystallinity, along with changes in apparent molecular-weight-related parameters, FTIR short-range order, and aggregate morphology. Molecular dynamics simulations showed progressively more negative local nonbonded interaction energies from HA to LA to AA, suggesting stronger local association tendencies for longer-chain fatty acids. Although AAM and BAM differed in matrix state, the chain-length-dependent ordering was preserved for key indicators. These findings suggest that guest chain length consistently influences RS5 assembly, while amylose matrix state affects the magnitude of structural changes.

## 1 Introduction

Starch research has evolved from fundamental structural studies to advanced functional applications (Chibh et al., 2024), with Resistant Starch Type 5 (RS5) emerging as a particularly valuable material due to its unique digestion resistance (Sun et al., 2021). RS5 is a typical starch inclusion complex defined by V-type crystallization, formed by encapsulating guest molecules within a cavity predominantly composed of amylose under suitable processing conditions (Zhou, Wang, et al., 2024; Zhou, Zheng, et al., 2024). Recent studies have shown that RS5 possesses tunable sustained-release properties, positioning it as a promising delivery vehicle for bioactive compounds (Wang et al., 2025). The fine structure of RS5 directly dictates its functional properties, including thermal stability and digestibility. Mechanistically, it is hypothesized that the compact microstructure and enhanced hydrophobicity of these inclusion complexes restrict enzyme accessibility, thereby conferring their characteristic resistance to digestion. Consequently, in-depth studies on the precise structural regulation of RS5 are crucial for advancing its application in the food, biomaterial, and pharmaceutical sectors.

The performance of RS5 in these applications is fundamentally determined by its molecular assembly process. Specifically, RS5 forms through the association of a host (amylose) and a guest (fatty acids) mediated primarily by hydrophobic forces, an interaction that has been extensively validated (Wang et al., 2020; Zhang, Hou, et al., 2024; Zhang, Li, & Fan, 2024; Zhang, Sun, et al., 2024). Preconditioning the amylose host, either by increasing its absolute content or exposing its binding sites, enhances fatty acid complexation, underscoring the host's essential role (Liu et al., 2023; Sun et al., 2023a). Concurrently, the guest's structural features—particularly the fatty acid chain length and degree of saturation—significantly influence RS5 formation, fine structure, and in vitro digestibility (Kong et al., 2019; Lu et al., 2021; Sun et al., 2021, Sun et al., 2023a, Sun et al., 2023b). Furthermore, Krishnan et al. (2021), Zhang, Hou, et al. (2024), Zhang, Li, and Fan (2024) and Zhang, Sun, et al. (2024) highlighted that mutual host-guest interactions and the apparent molecular weight of amylose critically alter the complex's bioavailability, warranting a comprehensive multiscale analysis of these assembly dynamics.

However, conventional macroscopic experimental techniques struggle to decouple the individual driving forces of complexation, such as hydrophobic interactions, steric hindrance, and hydrogen bonding. In contrast, molecular simulation techniques facilitate a meticulous examination of these localized intermolecular forces (Cheng et al., 2019; Gao et al., 2020, Gao et al., 2021). Despite this methodological advance, critical knowledge gaps persist regarding the interplay between molecular-level forces and macroscopic structural outcomes. For instance, Feng et al. (2024) reported that while distearoylglycerol (DSG) exhibits profound steric hindrance, it simultaneously possesses the strongest intermolecular binding forces, complicating structural predictions. Moreover, a comprehensive understanding of how the initial chain state (e.g., molecular weight and processing history) of the amylose couples with guest characteristics to influence RS5 formation remains elusive. A clearer comparative understanding of how local host–guest interaction tendencies relate to experimentally observed structural trends remains needed.

Our previous work using commercial high-amylose starches suggested that the apparent molecular state of the amylose may be associated with the digestive fate of RS5 (Zhang, Hou, et al., 2024; Zhang, Li, & Fan, 2024; Zhang, Sun, et al., 2024). However, because those comparisons involved starches with different structural backgrounds, the observed differences could not be attributed to a single factor. In the present study, rather than attempting to isolate one independent causal variable, we asked whether chain-length-dependent assembly trends could be consistently observed across two amylose matrices with different structural backgrounds. To address this question, two high-amylose matrices (both >91% amylose) prepared by fundamentally distinct processing routes—autoclaving and butanol recrystallization—were used as comparative model systems. Using these two high amylose matrices, we systematically prepared complexes with three saturated fatty acids of increasing chain length, namely hexanoic acid (C6), lauric acid (C12), and arachidic acid (C20). By combining gel permeation chromatography, UV–vis spectroscopy, fatty acid quantification, X-ray diffraction, Fourier transform infrared spectroscopy, scanning electron microscopy, laser diffraction, and molecular dynamics simulations, this study aimed to examine whether chain-length-dependent structural trends could be consistently observed across two distinct amylose matrices. In this context, the focus of the work is not to isolate a single causal factor, but to compare the similarity and consistency of structural responses under two different matrix backgrounds.

## 2 Materials and methods

### 2.1 Materials

Natural high-amylose cornstarch NF-CGK (amylose content of 87.35% ± 1.18, peak average molecular weight of 21,282 g/mol) and NF-G370CK (amylose content of 71.27% ± 2.20, peak average molecular weight of 188,044 g/mol) were from Henan Xinfuwang New Material Technology Co. (Luohe, China). Lipids (hexanoic acid, lauric acid, and arachidic acid), 1-butanol (99.5%), and DMSO (99%) were purchased from Shanghai Macklin Biochemical Technology Co., Ltd. (Shanghai, China); the water utilized was deionized water (Hangzhou Wahaha Group Co., Ltd., China). All other reagents were of analytical grade.

### 2.2 Preparation of two amylose matrices with comparable amylose contents

#### 2.2.1 Autoclaved NF-CGK

A total of 10 g of NF-CGK starch was dispersed in 90 mL of deionized water, thoroughly mixed, and subsequently subjected to autoclaving at 121 °C for 15 min. The processed mixture was then lyophilized to yield Autoclave-treated amylose matrix (AAM). The content of amylose was quantified via the Megazyme K-AMYL assay.

#### 2.2.2 High-amylose starch purified from NF-G370

Five grams of NF-G370CK starch was dissolved in 150 mL of DMSO solution and then placed in a full-temperature shaker at low speed for 24 h. The mixture was centrifuged at 2000 rpm for 15 min, and the supernatant was subsequently extracted. To this mixture, a volume of n-butanol that was twice that of the supernatant was added. The sample was allowed to stand at room temperature for 24 h. The mixture was subsequently centrifuged at 4000 rpm for 10 min, after which the precipitate was retained, which, at this stage, constituted the crude amylose.

A 1 g sample of crude amylose was dissolved in 50 mL of water saturated with 1-butanol. The solution was heated until it became transparent and then cooled to room temperature, after which it was heated to 2–4 °C and allowed to stand for 24 h. Centrifugation at 8000 rpm for 20 min was performed. The precipitate was subjected to this procedure three times. The mixture was then washed five times with anhydrous ethanol, followed by freeze-drying to yield amylose. The sample is designated Butanol-extracted amylose matrix (BAM). The amylose content was determined via Megazyme K-AMYL. The two preparation routes used to obtain amylose matrices inevitably resulted in differences in chain integrity and fine structure.

Throughout this study, the terms AAM and BAM refer to two distinct amylose matrices derived from different corn starch sources and prepared via different protocols. These procedures were necessary to achieve high and comparable amylose contents (>91%). These procedures were used to obtain matrices with similarly high amylose contents while preserving distinct apparent molecular states. Because differences in processing route inevitably affect chain integrity and fine structure, AAM and BAM are interpreted here as two model matrices with different preparation histories rather than as isolated molecular-weight variants. Both matrices were subsequently subjected to the same RVA treatment prior to complexation, allowing their responses to fatty acid chain length to be compared within a unified experimental framework.

#### 2.2.3 Amylose content determination

The amylose content was determined via the Megazyme K-AMYL (Amylose/Amylopectin Assay Kit). The formula for calculating the amylose content is as follows:

(1) $$\text{Amylose}\%\left(w/w\right)=\frac{\text{Absorbance}\;\left(\mathrm{Con}\;A\;\text{Supernatant}\right)}{\text{Absorbance}\;\left(\text{Total Starch Aliquot}\right)}\times \frac{6.15}{9.2}\times 100\;$$

6.15: Con A dilution factor; 9.2: Total starch dilution factor.

### 2.3 Preparation of amylose–lipid complexes

The methods of (Chao et al., 2018) were used with some modifications. The amylose was directly introduced into the RVA high-temperature aluminum canister. Three grams of amylose (AAM or BAM), 10% lipids (hexanoic acid, lauric acid, and arachidic acid, w/w), and 27 g of water were used. The sample was maintained at 50 °C for 2 min, heated to 130 °C and held for 1 min, followed by cooling to 50 °C and holding for 3 min. The stirring speed was set to 960 rpm for the initial 10 s, followed by continuous stirring at 160 rpm throughout the remainder of the process. Subsequently, the samples were washed five times with anhydrous ethanol to remove free fatty acids. Finally, the complexes were freeze-dried. We conducted digestion characteristic measurements on the complex, with the methods and results detailed in the Supplementary material.

### 2.4 Complex index (CI) of the amylose–lipid complexes

The complex index (CI) of the amylose–lipid complexes was determined and calculated according to the methods of (Zhang, Hou, et al., 2024; Zhang, Li, & Fan, 2024; Zhang, Sun, et al., 2024), all procedures were performed uniformly. The calculation formula was as follows:

(2) $$\mathrm{CI}\left(\%\right)=\frac{A_{0}-A_{1}}{A_{0}}\times 100\;$$

where A_0_ is the absorbance value of the control and A_1_ is the absorbance of the amylose–lipid complexes.

### 2.5 Molecular weight of amylose (gel permeation chromatography)

The samples were determined and calculated according to the methods of (Chang et al., 2018; Hu et al., 2022) with some modifications. All other procedures and conditions were consistent with those described in Zhang, Hou, et al. (2024), Zhang, Li, and Fan (2024) and Zhang, Sun, et al. (2024).

### 2.6 MD simulation

#### 2.6.1 Molecular docking

The construction of a glycan chain composed of 26 α-d-glucose units was performed via the Glycam website (http://glycam.org/). The simulation software used was Gromacs 2022.4 (Abraham et al., 2015), with the GLYCAM_06j-1 force field and the TIP3P water model. Energy minimization of the system was conducted in two steps: the first step utilized the steepest descent method (steep) for 10,000 iterations, and the second step employed the conjugate gradient (cg) method for 5000 iterations. The system was then equilibrated. The equilibration process consisted of two consecutive stages: the first stage involved 500 ps of NVT optimization at 303.15 K, followed by 500 ps of NPT optimization at 1 atm pressure in the second stage. After equilibration, a 50 ns production simulation was carried out, with the V-rescale (Bussi et al., 2007) algorithm for temperature control and the Parrinello–Rahman (Parrinello & Rahman, 1981) algorithm for pressure control. The simulation time step was set to 2 fs. The temperature was maintained at 303.15 K, and the pressure was 1 bar. Electrostatic interactions were calculated via the particle–mesh Ewald (PME) (Essmann et al., 1995) algorithm, with a cutoff radius of 12 Å for Coulomb and van der Waals interactions. Hydrogen bonds were constrained via the LINCS (Hess et al., 2008) algorithm.

Preprocessing of the glycan chain, arachidic acid, lauric acid, and n-hexanoic acid was performed via AutoDock Tools-1.5.6, and docking was conducted via AutoDock Vina 1.1.2 (Trott & Olson, 2010). The docking center coordinates for the glycan chain were set at (−0.061, −0.127, −0.041), with a grid size of 126 Å × 126 Å × 126 Å in the XYZ directions, and 10 docking attempts were made. The lowest binding energy conformations postdocking were analyzed via PyMOL v2.4.0a0 (PyMOL. Retrieved from http://www.pymol.org/pymol).

#### 2.6.2 Molecular dynamics simulation calculation

The construction of a linear amylose composed of 26 α-d-glucose units was performed via the glycam (http://glycam.org/) website. Topology files for arachidic acid, lauric acid, and n-hexanoic acid were generated via ACPYPE (Sousa Da Silva & Vranken, 2012) (https://bio2byte.be/acpype/) for subsequent kinetic simulations.

The simulation software used was Gromacs 2022.4 (Abraham et al., 2015). The force field for linear amylose was GLYCAM_06j-1, whereas the force fields for arachidic acid, lauric acid, and n-hexanoic acid were based on GAFF. The water model used was TIP3P. Energy minimization was conducted on the system, with the first step employing the steepest descent method (steep) for 10,000 iterations, followed by a conjugate gradient (cg) for 5000 iterations in the second step. The energy-optimized system was then subjected to equilibration. The equilibration process consisted of two consecutive stages: the first stage involved NVT optimization at 303.15 K for 500 ps, and the second stage involved NPT optimization at 1 atm for 500 ps. After equilibration, a production simulation of 50 ns was performed. The V-rescale temperature control algorithm was used (Bussi et al., 2007), and the pressure control algorithm was Parrinello-Rahman. The simulation time step was set to 2 fs. The temperature was maintained at 303.15 K, and the pressure was maintained at 1 bar. Electrostatic interactions were calculated via the particle–mesh Ewald (PME) algorithm, with a cutoff radius of 12 Å for both Coulombic and van der Waals interactions. Hydrogen bonds were constrained via the LINCS (Hess et al., 2008) algorithm.

The analysis of the kinetic simulation trajectory utilized built-in commands in Gromacs, whereas visualization and interaction analysis were conducted via PyMOL v2.4.0a0.

### 2.7 Quantification of fatty acids

Preprocessing:

(1) An appropriate amount of the sample was weighed and transferred to a 250 mL flat-bottom flask. Next, 2.0 mL of methanol, approximately 100 mg of pyrogallol, and a few boiling chips were added, followed by the addition of 2 mL of 95% ethanol and 4 mL of water. Mix thoroughly.
(2) Then, 10 mL of hydrochloric acid solution (8.3 mol/L) was added, the mixture was mixed well, and the mixture was placed in a water bath between 70 °C and 80 °C for hydrolysis for 40 min. The flask was shaken every 10 min to ensure that the particles adhering to the flask walls were mixed with the solution. After hydrolysis was complete, the flask was removed, and the mixture was allowed to cool to room temperature.
(3) For the hydrolyzed sample, 10 mL ethanol (95%) was added, and the mixture was mixed thoroughly. The hydrolyzed solution from the flask was transferred into a separatory funnel. The solution was extracted three times with a total of 150 mL of a mixture of diethyl ether and petroleum ether.

Instrument parameters:

(1) GCMS (Agilent 8860-5977B, Agilent Technologies, USA), chromatographic column information: Agilent 122–7032.
(2) UPLC–MS/MS (Waters TO-S Micro, Waters Corporation, USA) was used, and the chromatographic column information was as follows: Agilent 122–7032 HSS T3 2.5 μm × 3.0–150 mm.

### 2.8 X-ray diffraction

The crystalline patterns of the complexes were determined via an X-ray diffractometer, SmartLab SE (Rigaku Corp, Tokyo, Japan), at 40 kV and 40 mA with CuKα radiation (λ = 1.54056 Å). The XRD results were analyzed via Jade 9 software.

### 2.9 Fourier transform infrared spectroscopy

Fourier transform infrared spectroscopy (FTIR) of the amylose–lipid complexes was performed according to Liu, Guan, et al. (2024) and Liu, Luo, et al. (2024). The FTIR spectra of the complexes were recorded via FTIR spectroscopy (Bruker Corporation-INVENIO, America).

### 2.10 Scanning electron microscopy

To observe the morphology of the samples the granules were fixed, sputter coated with gold and imaged by scanning electron microscopy (SEM, Carl Zeiss AG-Sigma300). Energy Spectrum: Ultim Max Nanoanalysis - Xplore30. The operating conditions were as follows: accelerating voltage of 5 kV and amplification of 5 K× and 10 K× (Zhang, Hou, et al., 2024; Zhang, Li, & Fan, 2024; Zhang, Sun, et al., 2024).

### 2.11 Particle size distribution

The samples were determined and calculated according to (Zhang, Hou, et al., 2024; Zhang, Li, & Fan, 2024; Zhang, Sun, et al., 2024), with some modifications. The particle size of the experimental samples was determined via a Mastersizer 3000+ instrument. The wet method was employed for measurement, with a particle refractive index of 1.68, a particle absorption of 0.1, a dispersant as water, a dispersant refractive index of 1.33, a scattering model set to Mie, and an analysis model set to general. The obscuration was 5–20%.

### 2.12 Statistical analysis

All the data in this study were preprocessed in Excel, Parallel experiments were conducted with three replicates, plotted in Origin 2024 (Education Edition) and analyzed via ANOVA via SPSS 25.0 (Inc., Chicago, IL, USA).

## 3 Results and discussion

### 3.1 Determination of amylose

As shown in Table 1, the amylose contents of AAM and BAM were 91.71% and 92.12%, respectively, confirming that the two matrices were comparable in terms of amylose level. However, their distinct preparation histories resulted in marked differences in apparent molecular-weight-related parameters. This design allowed the two systems to be compared under similar amylose content but different initial matrix states. When complexed with fatty acids of different chain lengths, both AAM and BAM exhibited variations in complexing index (CI), lipid content (LC), Type-V crystallinity, and resistant starch (RS) content. Notably, although the absolute values differed between the two amylose matrices, the overall direction of change across HA, LA, and AA was broadly consistent. This observation indicates that amylose content alone was not the primary determinant of complexation efficiency; rather, the apparent molecular state and processing history of the amylose appear to exert a substantial influence, while the lipid chain-length-dependent trends remained discernible in both systems.

**Table 1:** Amylose content and selected complexation-related properties of the matrices and amylose–lipid complexes.

Sample	Content (%)	Mp (g/mol)	CI (%)	Type-V (%)	LC (mg/g)	RS (%)
AAM	91.71 ± 1.04	21,006 ± 276^c^	–	16.96 ± 1.01^d^	–	39.22 ± 3.83
AAM-HA	–	29,126 ± 0^b^	27.80 ± 1.32^b^	21.27 ± 1.51^c^	11.6	43.72 ± 4.42^c^
AAM-LA	–	30,498 ± 50^a^	33.37 ± 3.54^a^	24.59 ± 0.27^b^	28.68	46.43 ± 3.22^b^
AAM-AA	–	30,623 ± 25^a^	28.72 ± 2.29^ab^	27.48 ± 0.89^a^	49.01	50.70 ± 2.13^a^
BAM	92.12 ± 2.62	289,951 ± 5232^c^	–	16.77 ± 1.74^d^	–	21.25 ± 4.53^d^
BAM-HA	–	209,095 ± 4388^b^	40.31 ± 3.49^b^	25.66 ± 0.44^c^	17.98	30.89 ± 3.84^c^
BAM-LA	–	222,701 ± 2356^b^	41.97 ± 2.35^b^	30.85 ± 0.53^b^	48.85	33.36 ± 1.50^b^
BAM-AA	–	247,740 ± 2658^a^	54.04 ± 1.60^a^	35.61 ± 0.11^a^	66.82	35.59 ± 1.41^a^

CI: Complexing index, %; Type V: Relative amount of V-shaped crystals, %; LC: lipid content; RS, resistant starch after 120 min of in vitro digestion; different lowercase letters (a-d) indicate significant differences (*P* < 0.05).

### 3.2 Complex index analysis

According to Table 1, under identical preparation conditions, the CI values of the AAM-based complexes were 27.80%, 33.37%, and 28.72% for AAM-HA, AAM-LA, and AAM-AA, respectively, whereas those of the BAM-based complexes were 40.31%, 41.97%, and 54.04% for BAM-HA, BAM-LA, and BAM-AA, respectively. The Complexing Index (CI) is a classical and widely used parameter for confirming starch–lipid complex formation in starch-based systems (Lu et al., 2021). In the present study, the CI results support the formation of amylose–lipid complexes in all samples, consistent with previous reports (Feng et al., 2024). At the same time, the absolute CI values differed between the AAM and BAM systems, indicating that the two matrices showed different overall complexing responses under the present experimental conditions.

However, when the data were examined from a more quantitative perspective, CI did not show a simple correspondence with other structural indicators. For example, no significant difference was observed between the CI values of AAM-AA and AAM-LA (*P* > 0.05), although their lipid contents differed substantially; a similar pattern was also found between BAM-HA and BAM-LA. More broadly, within the present experimental framework, CI did not show a clear linear relationship with lipid content (LC), V-type crystallinity, short-range order (1050/1022), or the apparent GPC parameters. This inconsistency is likely related to the characteristics of the present high-amylose system rather than to a general invalidity of the method. As suggested by Feng et al. (2024), CI may not always accurately reflect the absolute extent of complexation, partly because iodine binding can be affected by competing structural changes such as retrogradation. Therefore, in this study, CI is interpreted primarily as a qualitative indicator confirming complex formation, whereas quantitative comparison of chain-length-dependent differences relies more appropriately on LC and XRD results.

### 3.3 Gel permeation chromatography

In our previous study (Zhang, Hou, et al., 2024; Zhang, Li, & Fan, 2024; Zhang, Sun, et al., 2024), the analysis mainly focused on the weight-average (Mw) and peak (Mp) molecular weights of the samples. In the present study, a more comprehensive GPC analysis was conducted to compare the apparent molecular-weight-related changes in amylose–lipid complexes formed with fatty acids of different chain lengths (Table 2).

**Table 2:** Molecular weights of the amylose–lipid complexes.

Sample	Mp (g/mol)	Mn (g/mol)	Mw (g/mol)	Mz (g/mol)	Mz + 1 (g/mol)	Polydispersity
AAM-HA	29,126 ± 0^b^	22,315 ± 92^b^	40,460 ± 40.5^b^	70,563 ± 47^b^	108,495 ± 182^b^	1.8188
AAM-LA	30,498 ± 50^a^	23,564 ± 319^a^	42,983 ± 204^a^	75,584 ± 107^a^	116,817 ± 95.5^a^	1.8082
AAM-AA	30,623 ± 25^a^	23,621 ± 136^a^	43,492 ± 91^a^	76,658 ± 174^a^	117,790 ± 115^a^	1.8302
BAM-HA	209,095 ± 4388^c^	140,131 ± 8082^c^	318,854 ± 10812^c^	602,228 ± 5778^c^	908,659 ± 18675^c^	1.9598
BAM-LA	222,701 ± 2356^b^	162,348 ± 3467^b^	360,583 ± 6207^b^	709,015 ± 15890^b^	1,087,711 ± 32741^b^	3.2120
BAM-AA	247,740 ± 2658^a^	177,953 ± 5228^a^	441,794 ± 4201^a^	908,766 ± 1552^a^	1,400,968 ± 11841^a^	3.4347

Mp, peak average molecular weight; Mn, number-average molecular weight; Mw, weight average molecular weight; Mz and Mz + 1, *Z*-average molecular weight. The polydispersity indicates the range of distribution of samples in the assay. Different lowercase letters (a-c) indicate significant differences (*P* < 0.05).

To account for chain degradation caused by high-temperature and shear treatment during preparation, the complexes were compared with their corresponding RVA-treated amylose benchmarks prepared under identical conditions but without added lipids (Table S3). The native BAM showed an initial Mp of 289,951 g/mol (Table 1), whereas the RVA-treated BAM benchmark showed a markedly lower Mp of 178,426 g/mol (Mw 238,596 g/mol; Mn 96,407 g/mol), indicating substantial degradation and fractionation during RVA treatment. Similarly, the RVA-treated AAM benchmark showed an Mp of 20,730 g/mol (Mw 33,850 g/mol; Mn 15,514 g/mol). These results indicate that thermal and shear treatment strongly affected the final GPC profiles of both matrices, which is consistent with previous reports showing that severe hydrothermal processing can induce chain scission and molecular redistribution in starch systems(Zavareze & Dias, 2011; Trinh, 2015).

Despite the clear differences between AAM and BAM in processing history and apparent molecular state, both systems showed the same overall chain-length-dependent trend after complexation, namely an increase in the apparent molecular-weight-related parameters from HA to LA to AA. In the AAM system, Mp increased from the RVA-treated benchmark to 29,126 g/mol for AAM-HA, 30,498 g/mol for AAM-LA, and 30,623 g/mol for AAM-AA. In the BAM system, the apparent Mp increased from 178,426 g/mol in the RVA-treated benchmark to 209,095 g/mol for BAM-HA, 222,701 g/mol for BAM-LA, and 247,740 g/mol for BAM-AA. Thus, although the absolute values differed between the two matrices, the direction of the chain-length-dependent change was consistent. This ordering is also in line with the trend observed in our previous study, in which starches from different sources subjected to the same treatment showed comparable molecular-weight-related changes after complexation (Zhang, Hou, et al., 2024; Zhang, Li, & Fan, 2024; Zhang, Sun, et al., 2024).

A noteworthy difference was observed in the extent of molecular-weight change. In the AAM system, the apparent molecular-weight-related parameters of AAM-LA and AAM-AA were very close, with most showing no significant difference, indicating that the increase from LA to AA was limited. In contrast, in the BAM system, BAM-AA remained significantly higher than BAM-LA across Mp, Mn, Mw, Mz, and Mz + 1, indicating a larger difference between LA and AA. This suggests that although both matrices followed the same overall trend (HA < LA < AA), the extent of molecular-weight change differed between them, with a relatively small difference between AAM-LA and AAM-AA but a more pronounced difference between BAM-LA and BAM-AA.

When these findings are considered together with our previous study (Zhang, Hou, et al., 2024; Zhang, Li, & Fan, 2024; Zhang, Sun, et al., 2024), an important implication emerges. Similar molecular-weight-related trends were observed not only across the two differently processed matrices examined here, but also across starches of different origins subjected to a common treatment in our earlier work. This cross-study consistency suggests that amylose molecular-weight-related characteristics may contribute importantly to the apparent GPC changes after complexation, alongside processing history and matrix state. However, because the present design does not isolate molecular weight and processing history as fully independent variables, this interpretation should still be regarded as comparative and associative rather than strictly causal.

To further examine the possible origin of these molecular-weight-related variations, we estimated the mass contribution of the retained fatty acids using two calculation approaches (Tables S3–S5). The results indicate that the retained fatty acid mass accounts for only part of the observed increase in the apparent molecular-weight-related parameters, suggesting that additional factors are involved. Under the present measurement conditions, the observed GPC changes may reflect not only the mass contribution of retained fatty acids, but also changes in the apparent association state and/or solution conformation of amylose after complexation (Yao et al., 2023). However, the present data do not allow these possibilities to be distinguished clearly. In this context, the MD results are considered only as supportive comparative information showing stronger local association tendencies for longer-chain fatty acids within the model system, rather than as direct evidence for aggregate formation or hydrodynamic volume changes in the actual GPC system. Therefore, the GPC results in this study are interpreted primarily as comparative evidence showing that, despite their different matrix backgrounds, both AAM and BAM exhibited a similar chain-length-dependent ordering in apparent molecular-weight-related changes after complexation.

### 3.4 Intermolecular forces and binding energy

To obtain a simplified view of local host–guest association, molecular docking and MD simulations were performed using a 26-glucose amylose helix and three fatty acids in water. This model was not intended to reproduce the polymer-scale behavior of the experimental AAM and BAM matrices. Instead, it was used only to compare relative local nonbonded interaction tendencies within a unified model environment.

**Fig. 1:**
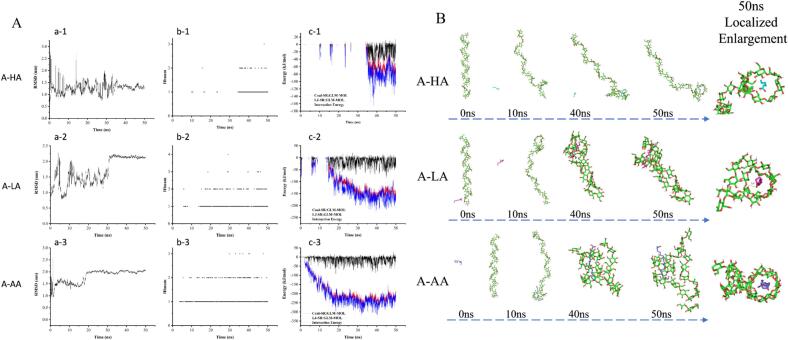
Root mean square deviation (RMSD), hydrogen bonds and intermolecular forces of the amylose–lipid complexes (A). Conformational changes based on snapshots during complexation between amylose and different lipids over a simulation period of 50 ns (B).

As shown in Fig. 1A, the RMSD values became relatively stable during the later stage of the simulation, and the 40–50 ns interval was therefore selected for comparative analysis. Representative final configurations are shown in Fig. 1B, indicating close association between the fatty acids and the amylose helix. However, particularly for arachidic acid (AA), the simulated configuration should be interpreted cautiously, because it may reflect partial insertion together with extra-cavity hydrophobic contacts rather than complete inclusion within the helix. The binding energies (BE) and mean interaction energies during the 40–50 ns period are presented in Table 3. For amylose and hexanoic acid (HA), the Coul-SR and LJ-SR values were − 15.44 and − 63.55 kJ/mol, respectively, giving a total mean interaction energy of −78.99 kJ/mol (BE: −2.6 kcal/mol). For lauric acid (LA), the corresponding values were − 17.74 and − 142.58 kJ/mol, yielding a total mean interaction energy of −160.32 kJ/mol (BE: −2.85 kcal/mol). For arachidic acid (AA), the Coul-SR and LJ-SR values were − 13.10 and − 225.54 kJ/mol, respectively, resulting in a total mean interaction energy of −238.74 kJ/mol (BE: −3.1 kcal/mol). These results indicate that the Lennard–Jones short-range term contributed most strongly to the observed differences among the three fatty acids, whereas Coulombic interactions made a smaller contribution. The ordering HA < LA < AA therefore suggests that, within this model, longer-chain fatty acids exhibited stronger local nonbonded association with the amylose helix than shorter-chain fatty acids. Similar lipid chain-length-dependent tendencies have also been reported in previous studies of starch–guest interactions (Cheng et al., 2019; Feng et al., 2024).

**Table 3:** Intermolecular forces and binding energies of the amylose–lipid complexes.

Sample	Lipid Mw (g/mol)	BE (kcal/mol)	Coul-SR (kJ/mol)	LJ-SR (kJ/mol)	Total (kJ/mol)
Amylose-HA	116.16	−2.6	−15.44	−63.55	−78.99
Amylose -LA	200.32	−2.85	−17.74	−142.58	−160.32
Amylose-AA	312.53	−3.1	−13.1	−225.54	−238.74

BE: binding energy; Coul-SR: Coulombic interaction energy; LJ-SR: Lennard–Jones short-range; Total: total average interaction energy. Lipid Mw: lipid molecular mass, HA: 116.16 g/mol; LA: 200.32 g/mol; AA: 312.53 g/mol.

Within this framework, the model-derived interaction energies became progressively more negative from HA to LA to AA (Table 3). These values are interpreted here only as comparative descriptors within the same model system, rather than as definitive binding energies for the actual macromolecular RS5 matrices. Notably, the experimental data in both AAM and BAM showed the same overall ordering from HA to LA to AA for lipid content and V-type crystallinity. A similar ordering was also observed for the apparent molecular-weight-related parameters in the GPC analysis. Thus, although the MD model does not directly describe the bulk structure of the experimental systems, the chain-length-dependent ordering observed in the simulation was consistent with the ordering of multiple experimental indicators. In this study, the MD results are therefore used as supportive comparative information for local host–guest association, rather than as direct evidence linking molecular-level interactions to bulk structural organization.

### 3.5 Quantitative determination of lipids

The lipid content (LC) was quantitatively analyzed (Table 1). In both the AAM and BAM systems, LC increased with fatty acid chain length, with complexes formed with arachidic acid (AA) showing the highest values, followed by those formed with lauric acid (LA) and hexanoic acid (HA). Specifically, AA-containing complexes exhibited LC values of 49.01 mg/g in AAM and 66.82 mg/g in BAM, both significantly higher than those of the corresponding LA- and HA-containing complexes (*P* < 0.05). These results indicate that the influence of guest chain length on lipid retention was consistently observed in both matrices.

The higher LC values observed for longer-chain fatty acids-complexes are consistent with the stronger local association tendency indicated by the MD results and with previous reports showing enhanced starch–guest interactions for longer-chain fatty acids (Feng et al., 2024). At the same time, solvent-related retention effects should also be considered, because the lower solubility of longer-chain fatty acids may facilitate their retention during the post-washing process (Sun et al., 2021). Therefore, the present LC results likely reflect the combined influence of guest chain length and amylose-related retention behavior under the current preparation and washing conditions. Notably, the chain-length-dependent ordering of LC was also consistent with the ordering observed in the GPC analysis, in which the apparent molecular-weight-related parameters increased from HA to LA to AA in both matrices. Although these two datasets describe different aspects of the complexes, their consistent ordering provides mutually supportive evidence that guest chain length was an important factor influencing complex formation in both AAM and BAM.

A comparison between the two complexes further showed that BAM consistently exhibited higher LC values than AAM for all three fatty acids (Table 1). This indicates that the two amylose matrices differed in the extent of lipid retention under the same experimental framework. This dependent difference was also broadly consistent with the GPC results, where BAM generally showed larger molecular-weight-related changes than AAM, particularly between LA and AA. Because AAM and BAM differ in processing history and apparent molecular state. When considered together with previous findings that amylose molecular characteristics can influence the complexing state of starch–lipid systems (Wang et al., 2025), the present data suggest that host amylose state may contribute to differences in lipid retention. Nevertheless, the overall chain-length-dependent ordering of LC (HA < LA < AA) was preserved in both systems, again indicating a similar directional influence of guest chain length across the two amylose backgrounds.

Finally, no significant correspondence was observed between the Complex Index (CI) values and the quantitative LC results (Table 1). This further supports the limitation of the iodine-binding method discussed in Section 3.2, indicating that although CI can confirm the presence of complex formation, it does not reliably reflect the amount of fatty acid retained in the complexes, particularly when comparing guest molecules with different chain lengths.

### 3.6 Crystalline structure

**Fig. 2:**
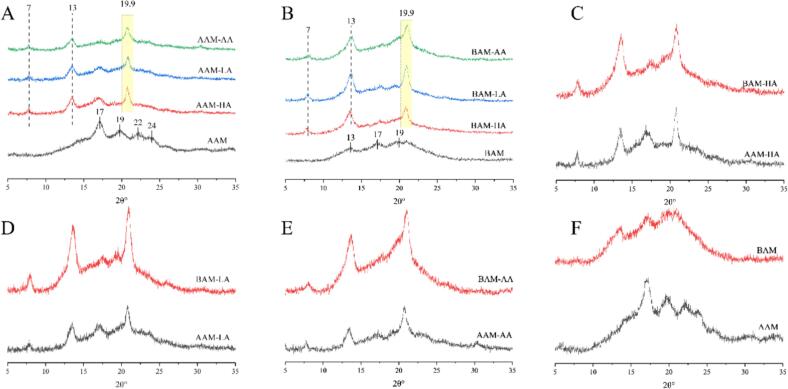
X-ray diffraction (XRD) patterns of amylose and amylose–lipid complexes. HA, LA, AA (hexanoic acid, lauric acid, arachidic acid).

The crystalline structures of the samples were analyzed via XRD (Fig. 2). Both the AAM-lipid and BAM-lipid complexes exhibited characteristic diffraction peaks at 2θ angles of approximately 7°, 13°, and 20°, confirming the formation of V-type crystalline polymorphs (Fig. 2A and B). While minor V-type peaks were also observed in the native amylose samples (AAM and BAM), likely stemming from endogenous lipids, the complexes displayed significantly sharper and more intense peaks within the characteristic regions (highlighted in yellow in Fig. 2).These diffraction features are generally consistent with the formation of Type II V-complexes reported in previous starch–lipid systems.

Quantitative analysis (Table 1) further showed that V-type crystallinity increased with fatty acid chain length in both matrices. For the AAM system, the V-type crystallinity increased significantly from 21.27% (HA) to 27.48% (AA) (*P* < 0.05). A similar trend was observed in the BAM system, where crystallinity rose from 25.66% (HA) to 35.61% (AA) (P < 0.05). Notably, this ordering was also consistent with the LC results described in Section 3.5. As shown in Table 1 and Table 3, the lipid loading capacity increased with chain length (HA < LA < AA), which corresponds with the increase in V-type crystallinity. Although crystallinity and LC describe different aspects of the complexes, their consistent chain-length-dependent ordering provides mutually supportive evidence that guest chain length was an important factor influencing the formation state of the complexes in both matrices.

Therefore, in the present system, V-type crystallinity appeared to be more informative than the Complex Index (CI) for comparing differences among complexes formed with fatty acids of different chain lengths. However, the present data support a comparative association rather than a strict quantitative proportional relationship between crystallinity and guest incorporation.

### 3.7 FTIR analysis

**Fig. 3:**
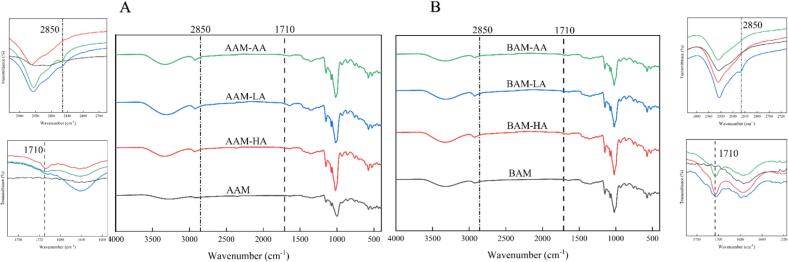
Fourier transform infrared (FTIR) spectroscopy of amylose and amylose–lipid complexes.

The FTIR spectra (Fig. 3) confirmed the formation of starch–lipid complexes, as indicated by the characteristic peaks at 1710 cm^−1^ and 2850 cm^−1^ in the complex samples (Kang et al., 2022). To further examine short-range structural features, the deconvoluted spectral ratios of 1050/1022 cm^−1^ and 1022/995 cm^−1^ were analyzed (Table S1). In general, the 1050/1022 cm^−1^ ratio, which is commonly used as an indicator of short-range order in starch-based systems, was lower in the complexes than in the corresponding amylose samples (AAM and BAM). This difference is consistent with previous reports and may be related to structural reorganization during processing and complexation (Zhou, Wang, et al., 2024; Zhou, Zheng, et al., 2024).

Within both the AAM and BAM systems, the 1050/1022 cm^−1^ ratio increased from HA to AA. Specifically, the ratio for AAM complexes increased from 1.13 in AAM-HA to 1.30 in AAM-AA, while that for BAM complexes increased from 0.89 in BAM-HA to 0.99 in BAM-AA. This ordering was also consistent with the chain-length-dependent ordering observed for the model-derived interaction energies, in which the average nonbonded interaction energies became progressively more negative from HA to LA to AA. Although the MD model does not directly describe the bulk short-range order of the experimental matrices, the similar HA < LA < AA ordering provides supportive comparative evidence that guest chain length was associated with consistent changes in local association tendency and FTIR-derived structural indicators. This ordering was also broadly consistent with the trends observed for LC and V-type crystallinity.

A further difference was observed between the two high amylose matrices: the 1050/1022 values of the BAM complexes (0.89–0.99) were lower than those of the corresponding AAM complexes (1.13–1.30), even though BAM exhibited a higher overall lipid-loading capacity (LC). This result indicates that the two matrices differed in the relationship between lipid retention and FTIR-derived short-range order under the present experimental conditions.

The FTIR results may also be considered together with the particle-related parameters shown in Table 4. Complexes formed with longer-chain fatty acids generally showed higher SSA values than those formed with shorter-chain fatty acids. Taken together with previous comparative studies on starch structural order (Liu, Guan, et al., 2024; Liu, Luo, et al., 2024; Maibam et al., 2023; Raza et al., 2023), the present results support the view that guest chain length was associated with consistent changes in short-range structural indicators across both matrices.

**Table 4:** Specific surface area, helical structure and short-range order of the amylose–lipid complexes.

Sample	Specific surface area (m^2^/kg)	1050/1022	1022/995
AAM-HA	775.90 ± 5.49^c^	1.13 ± 0.01^c^	1.10 ± 0.01^ab^
AAM-LA	839.00 ± 9.34^b^	1.16 ± 0.05^c^	1.15 ± 0.03^a^
AAM-AA	972.10 ± 17.93^a^	1.30 ± 0.02^b^	1.03 ± 0.03^c^
BAM-HA	112.10 ± 2.77^c^	0.89 ± 0.01^c^	1.31 ± 0.02^b^
BAM-LA	175.90 ± 6.07^b^	0.88 ± 0.01^c^	1.41 ± 0.01^a^
BAM-AA	197.00 ± 7.82^a^	0.99 ± 0.03^a^	1.24 ± 0.01^c^

SSA: Specific surface area; significance analysis of columns a, b, c; different lowercase letters (a-c) indicate significant differences (*P* < 0.05).

### 3.8 Scanning electron microscopy analysis

**Fig. 4:**
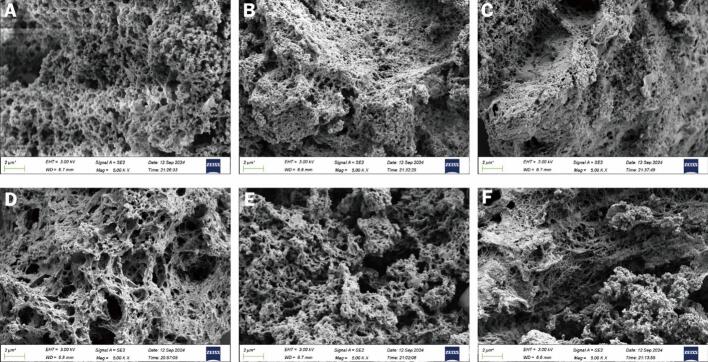
Scanning electron micrographs (SEM) of the samples. (A–C) AAM complexes with HA, LA, and AA; (D–F) BAM complexes with HA, LA, and AA. (Magnification: 5000×).

The morphology of the freeze-dried complexes was characterized via scanning electron microscopy (SEM) (Fig. 4). The SEM micrographs revealed that the samples assembled into continuous, three-dimensional stacked aggregates. Crucially, the overall microscopic morphology exhibited a distinct dependence on the fatty acid chain length. Complexes formed with short-chain fatty acids (e.g., AAM-HA and BAM-HA) displayed a relatively loose microscopic aggregation, characterized by a coarser surface framework and less dense packing. Conversely, as the chain length increased to lauric acid (LA) and arachidic acid (AA), the structure underwent significant densification. Consequently, the AAM-AA and BAM-AA complexes exhibited a highly compact and continuous microscopic morphology, characterized by tighter granular fusion and a significantly denser surface texture. As corroborated by the localized molecular dynamics (MD) simulations (Section 3.4), arachidic acid (AA) exhibited the strongest intermolecular binding energy with amylose (−238.74 kJ/mol) compared to hexanoic acid (HA, −78.99 kJ/mol). This is consistent with our previous research findings (Zhang, Hou, et al., 2024; Zhang, Li, & Fan, 2024; Zhang, Sun, et al., 2024). The SEM observations indicate that complexes formed with longer-chain fatty acids showed denser aggregate appearance than those formed with shorter-chain fatty acids. This ordering was also broadly consistent with the ordering observed in the MD model, although the latter is interpreted only as supportive comparative information at the local interaction level.

### 3.9 Particle size distribution analysis

The specific surface area (SSA) values derived from laser diffraction are reported here only as comparative particle-related parameters under the measurement conditions. Although higher calculated SSA values are generally associated with smaller apparent particle sizes, these values should be interpreted cautiously for irregular aggregated particles, as they do not directly quantify internal surface area, porosity, or packing density.

Compared with the corresponding native matrices (Table S6), the calculated SSA values of the AAM- and BAM-based complexes were higher (Table 4, P < 0.05). Together with the SEM observations (Section 3.8), these data indicate differences in the apparent aggregation state and particle-related characteristics among samples. However, these measurements are descriptive and do not by themselves resolve the detailed mode of guest retention or internal structural organization. Therefore, in the present study, the SSA and apparent particle size data are interpreted only as comparative bulk-level descriptors, and no direct causal relationship is inferred between these parameters and the localized interaction energies obtained from the MD model.

### 3.10 Factors influencing RS5 formation: Amylose state and guest chain length

**Fig. 5:**
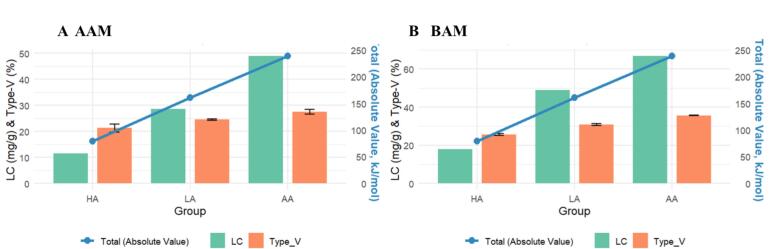
Parallel trends between model-derived interaction strength and selected experimental indicators. The bar charts represent the experimental Lipid Content (LC) and V-type crystallinity (referenced to the left axis), while the Blue solid line represents the magnitude of the total intermolecular interaction energy derived from MD simulations (referenced to the right axis). (A) AAM complexes; (B) BAM complexes. The similar HA < LA < AA ordering observed in LC, V-type crystallinity, and model-derived interaction energies is presented here as comparative evidence only. These data do not establish a direct causal link between local model interactions and bulk structural organization. (For interpretation of the references to colour in this figure legend, the reader is referred to the web version of this article.)

Taken together, the present results indicate that both the initial matrix state of amylose and the chain length of the fatty acid guest were associated with measurable differences in the structural features of the resulting RS5 complexes. Under the present experimental conditions, BAM consistently showed higher lipid content than AAM, suggesting that the initial molecular state and processing history of the host amylose influenced the overall complexation outcome. Across both matrices, increasing fatty acid chain length was accompanied by higher lipid content and higher V-type crystallinity, together with differences in FTIR short-range order, aggregate appearance in SEM, and particle-related parameters derived from laser diffraction (See Fig. 5).

The MD analysis further showed that, within a 26-glucose single-helix model, longer-chain fatty acids exhibited more negative local nonbonded interaction energies than shorter-chain fatty acids. These model results provide supportive information on relative local association tendencies within a simplified host–guest environment. However, they do not constitute direct evidence for the bulk structural organization of the experimental AAM and BAM systems, nor do they establish a causal mechanistic link to crystallinity, aggregate morphology, or particle-related parameters.

Specifically, guest chain length was systematically associated with differences in lipid retention and structural descriptors, and these experimental trends were broadly consistent with the ordering observed in the MD model. Within the evidential limits of the current dataset, this consistency suggests that local host–guest association may contribute to the observed assembly behavior. Overall, the main conclusion of this section is that RS5 assembly in the present system depends on both host amylose state and guest chain length, as demonstrated by convergent experimental trends across multiple characterization methods.

## 4 Conclusion

This study compared the assembly behavior of resistant starch type 5 (RS5) complexes in two amylose-rich systems, AAM and BAM, which differed in processing history, and molecular-weight-related characteristics. Under the present experimental conditions, both systems showed the same overall chain-length-dependent ordering across multiple structural indicators, with HA < LA < AA observed for lipid content, V-type crystallinity, FTIR-derived short-range order, and apparent molecular-weight-related parameters. These results indicate that guest chain length exerted a similar directional influence on RS5 formation in both systems despite their different structural backgrounds.

At the same time, the two systems differed in the extent of these changes. BAM generally exhibited higher lipid retention and larger molecular-weight-related differences than AAM, whereas AAM and BAM also differed in their FTIR-derived short-range order under the same experimental framework. These findings suggest that, although the overall chain-length-dependent trends were preserved, the magnitude and detailed expression of the structural changes were influenced by differences in processing history and molecular-weight-related characteristics. The MD model showed the same HA < LA < AA ordering in local nonbonded interaction energies, providing supportive comparative information for the effect of guest chain length at the model level. However, these model-derived results are interpreted here only as comparative descriptors of local association tendency, rather than as direct evidence for the bulk structural organization of the experimental systems. Methodologically, the present results also indicate that V-type crystallinity was more informative than the Complex Index (CI) for comparing differences among complexes formed with fatty acids of different chain lengths, whereas CI mainly served as a qualitative indicator of complex formation. Overall, this study provides comparative experimental evidence that two amylose-rich systems with distinct processing histories and molecular-weight-related characteristics can exhibit similar chain-length-dependent assembly trends during RS5 formation, while differing in the extent of the associated structural changes.

## CRediT authorship contribution statement

**Mingyi Zhang:** Writing – review & editing, Writing – original draft, Methodology, Investigation, Formal analysis, Data curation, Conceptualization. **Caiming Li:** Methodology, Investigation, Conceptualization. **Jihong Huang:** Supervision, Resources, Funding acquisition, Conceptualization. **Jingbo Zhou:** Supervision, Resources, Funding acquisition, Conceptualization.

## Declaration of competing interest

The authors declare that they have no known competing financial interests or personal relationships that could have appeared to influence the work reported in this paper.

## Data Availability

Data will be made available on request.

## Appendix A Supplementary data

Supplementary material Provides method for RS digestion; tables on complex structure, molecular weight, particle size; analysis that fatty acid chain length, not just mass, drives RS5 assembly.
