# Supplementary material for: Assembly of resistant starch type 5 in two amylose matrices: effects of fatty acid chain length and matrix background on structural characteristics
Source: Food Chem X. 2026 May 2;36:103942. doi: 10.1016/j.fochx.2026.103942 (PMC13217497; doi:10.1016/j.fochx.2026.103942)
Supplement: Supplementary material — Provides method for RS digestion; tables on complex structure, molecular weight, particle size; analysis that fatty acid chain length, not just mass, drives RS5 assembly. [file mmc1.docx]

**Assembly of Resistant Starch Type 5 in Two Amylose Matrices: Effects of Fatty Acid Chain Length and Matrix Background on Structural Characteristics**

Mingyi Zhang^1^, Caiming Li^1^, Jihong Huang^3,*^, Jingbo Zhou^2,*^

^1^State Key Laboratory of Food Science and Resources, Jiangnan University, Wuxi, Jiangsu 214122, China.

^2^Rice Research Institute, Guangdong Academy of Agricultural Sciences, Guangzhou, 510642, China.

^3^State Key Laboratory of Crop Stress Adaptation and Improvement College of Agriculture, Henan University, Kaifeng, 475004, China.

* Corresponding author:

Email addresses: huangjh@henu.edu.cn (J.H. Huang); zhoujingbo@gdaas.cn (J.B. Zhou).

# 2 Materials and methods

**2.2.3 *In vitro* digestion property**

Two hundred milligrams of starch sample was added to a 50 ml centrifuge tube, 15 ml of sodium acetate acetate buffer (pH 5.2, 0.2 mol/L) was added, the tube was incubated at 100°C for 60 min, and the tube was cooled to room temperature. Then, 5 ml of enzyme mixture (290 U/mL amylase, 20 U/ml saccharase) was added. In the end, 0.1 ml of enzyme solution was added to 0.9 ml of anhydrous ethanol to inactivate the enzyme. The samples were subjected to 0, 20, 40, 60 and 120 min of enzyme digestion, and the absorbance at 505 nm was determined after the addition of GOPOD reagent. All three parallel tests were carried out in this study. The results were calculated by using the following formula:

$$RDS \left( \% \right)=\left( G_{20}-G_{0} \right)\times\frac{0.9}{TS}\times100 (1)$$

$$SDS \left( \% \right)=\left( G_{120}-G_{20} \right)\times\frac{0.9}{TS}\times100 (2)$$

$$RS \left( \% \right)=TS-RDS-SDS (3)$$

$$Hydrolysis rate \left( \% \right)=G_{t}\times\frac{0.9}{TS}\times100 (4)$$

where G_0_, G_20_, G_120_, and G_t_ represent the amounts of glucose at 0, 20, 120, and t min, respectively, and TS indicates the total starch amount.

**Table S1** Complex indices, crystalline structures, helical structures and short-range orders of the amylose‒lipid complexes

|  | CI (%) | Type-V (%) | 1050/1022 | 1022/995 | LC (mg/g) | RS (%) |
| --- | --- | --- | --- | --- | --- | --- |
| AAM | -- | 16.96 ± 1.01^d^ | 1.68 ± 0.05^a^ | 1.08 ± 0.02^ab^ | -- | -- |
| AAM-HA | 27.80 ± 1.32^b^ | 21.27 ± 1.51^c^ | 1.13 ± 0.01^c^ | 1.10 ± 0.01^ab^ | 11.6 | 43.72 ± 4.42^c^ |
| AAM-LA | 33.37 ± 3.54^a^ | 24.59 ± 0.27^b^ | 1.16 ± 0.05^c^ | 1.15 ± 0.03^a^ | 28.68 | 46.43 ± 3.22^b^ |
| AAM-AA | 28.72 ± 2.29^ab^ | 27.48 ± 0.89^a^ | 1.30 ± 0.02^b^ | 1.03 ± 0.03^c^ | 49.01 | 50.70 ± 2.13^a^ |
| BAM | -- | 16.77 ± 1.74^d^ | 0.95 ± 0.03^b^ | 1.18 ± 0.04 ^d^ | -- | -- |
| BAM-HA | 40.31 ± 3.49^c^ | 25.66 ± 0.44^c^ | 0.89 ± 0.01^c^ | 1.31 ± 0.02^b^ | 17.98 | 30.89 ± 3.84^c^ |
| BAM-LA | 41.97 ± 2.35^b^ | 30.85 ± 0.53^b^ | 0.88 ± 0.01^c^ | 1.41 ± 0.01^a^ | 48.85 | 33.36 ± 1.50^b^ |
| BAM-AA | 54.04 ± 1.60^a^ | 35.61 ± 0.11^a^ | 0.99 ± 0.03^a^ | 1.22 ± 0.01^c^ | 66.82 | 35.59 ± 1.41^a^ |

LC: lipid content; RS, resistant starch after 120 minutes of *in vitro* digestion; Different lowercase letters (a-d) indicate significant differences, (P < 0.05).

**Table S2** Molecular weights of the amylose‒lipid complexes

| Sample | Mp (g/mol) | Mn (g/mol) | Mw (g/mol) | Mz (g/mol) | Mz+1 (g/mol) | Polydispersity |
| --- | --- | --- | --- | --- | --- | --- |
| AAM | 21,006 ± 276^c^ | 15,227 ± 287^c^ | 33,139 ± 710^d^ | 68,911 ± 2498^b^ | 119,306 ± 5132^a^ | 2.1819 |
| AAM-HA | 29,126 ± 0^b^ | 22,315 ± 92^b^ | 40,460 ± 40.5^c^ | 70,563 ± 47^b^ | 108,495 ± 182^b^ | 1.8188 |
| AAM-LA | 30,498 ± 50^a^ | 23,564 ± 319^a^ | 42,983 ± 204^a^ | 75,584 ± 107^a^ | 116,817 ± 95.5^a^ | 1.8082 |
| AAM-AA | 30,623 ± 25^a^ | 23,621 ± 136^a^ | 43,492 ± 91^a^ | 76,658 ± 174^a^ | 117,790 ± 115^a^ | 1.8302 |
| BAM | 289,951 ± 5232^c^ | 168,096 ± 7178^ab^ | 780,204 ± 15051^a^ | 620,237 ± 1260^d^ | 5,168,373 ± 348752^a^ | 4.1897 |
| BAM-HA | 209,095 ± 4388^b^ | 140,131 ± 8082^b^ | 318,854 ± 10812^d^ | 602,228 ± 5778^c^ | 908,659 ± 18675^b^ | 1.9598 |
| BAM-LA | 222,701 ± 2356^b^ | 162,348 ± 3467^ab^ | 360,583 ± 6207^c^ | 709,015 ± 15890^b^ | 1,087,711 ± 32741^b^ | 2.2120 |
| BAM-AA | 247,740 ± 2658^a^ | 177,953 ± 5228^a^ | 441,794 ± 4201^b^ | 908,766 ± 1552^a^ | 1,400,968 ± 11841^b^ | 2.4347 |

Mp, peak average molecular weight; Mn, number-average molecular weight; Mw, weight average molecular weight; Mz and Mz+1, Z-average molecular weight. The polydispersity indicates the range of distribution of samples in the assay. Different lowercase letters (a-c) indicate significant differences (P < 0.05).

**Table S3 Amylose content and Molecular weight of amylose after RVA treatment**

| Sample | Content (%) | Mp (g/mol) | Mn (g/mol) | Mw (g/mol) | Mz (g/mol) | Mz+1 (g/mol) | Polydispersity |
| --- | --- | --- | --- | --- | --- | --- | --- |
| AAM | 92.11 ± 2.04 | 20,730 | 15,514 | 33,850 | 71,410 | 124,439 | 2.181900 |
| BAM | 90.11 ± 1.39 | 178,426 | 96,407 | 238,596 | 440,848 | 645683 | 2.474883 |

**Table S4-1 Calculation of AAM Molecular Weight Based on Fatty Acid Content**

| Index | Sample | LC(mg/g) | TΔM (g/mol) | AM(g/mol) | AΔM (g/mol) | Proportion |
| --- | --- | --- | --- | --- | --- | --- |
| Mn: | BAM-HA | 11.60 | 182.1 | 22315 | 6,801 | 2.68% |
|  | BAM-LA | 28.68 | 458.1 | 23,564 | 8,050 | 5.69% |
|  | BAM-AA | 49.01 | 799.5 | 23,621 | 8,107 | 9.86% |
| Mw | BAM-HA | 11.60 | 397.3 | 40460 | 6610 | 6.01% |
|  | BAM-LA | 28.68 | 999.5 | 42,983 | 9,133 | 10.94% |
|  | BAM-AA | 49.01 | 1,744.50 | 43,492 | 9,642 | 18.09% |
| Mp | BAM-HA | 11.60 | 243.3 | 29126 | 8396 | 2.9% |
|  | BAM-LA | 28.68 | 612.1 | 30,498 | 9,768 | 6.27% |
|  | BAM-AA | 49.01 | 1,068.30 | 30,623 | 9,893 | 10.80% |

The benchmark for AAM, Mp 20730 g/mol, Mw 33850 g/mol, Mn 15,514 g/mol, LC: lipid content,T ΔM, theoretical molecular weight increment, AM, actual measured molecular weight, AΔM, actual molecular weight increment.

**Table S4-2 Calculation of BAM Molecular Weight Based on Fatty Acid Content**

| Index | Sample | LC (mg/g) | TΔM (g/mol) | AM(g/mol) | AΔM (g/mol) | Proportion |
| --- | --- | --- | --- | --- | --- | --- |
| Mn | BAM-HA | 17.98 | 1,765 | 140,131 | 43,724 | 4.0% |
|  | BAM-LA | 48.85 | 4,951 | 162,348 | 65,941 | 7.5% |
|  | BAM-AA | 66.82 | 6,903 | 177,953 | 81,546 | 8.5% |
| Mw | BAM-HA | 17.98 | 4,368.5 | 318,854 | 80,258 | 5.44% |
|  | BAM-LA | 48.85 | 12,254.0 | 360,583 | 121,987 | 10.05% |
|  | BAM-AA | 66.82 | 17,084.6 | 441,794 | 203,198 | 8.41% |
| Mp | BAM-HA | 17.98 | 3,266.8 | 209,095 | 30,669 | 10.65% |
|  | BAM-LA | 48.85 | 9,163.8 | 222,701 | 44,275 | 20.70% |
|  | BAM-AA | 66.82 | 12,776.1 | 247,740 | 69,314 | 18.43% |

The benchmark of BAM: Mp 178426 g/mol, Mw 238596 g/mol, Mn 96407 g/mol

**Table S5-1 Calculation of AAM Molecular Weight Based on Reaction Conditions**

| Index | Sample | TΔM (g/mol) | AΔM (g/mol) | TΔM/AΔM |
| --- | --- | --- | --- | --- |
| Mn | BAM-HA | 1,551.40 | 6,801 | 22.81% |
|  | BAM-LA | 1,551.40 | 8,050 | 19.27% |
|  | BAM-AA | 1,551.40 | 8,107 | 19.14% |
| Mw | BAM-HA | 3,385.00 | 6,610 | 51.21% |
|  | BAM-LA | 3,385.00 | 9,133 | 37.06% |
|  | BAM-AA | 3,385.00 | 9,642 | 35.11% |
| Mp | BAM-HA | 2,073.00 | 8,396 | 24.69% |
|  | BAM-LA | 2,073.00 | 9,768 | 21.22% |
|  | BAM-AA | 2,073.00 | 9,893 | 20.95% |

**Table S5-2 Calculation of BAM Molecular Weight Based on Reaction Conditions**

| Index | Sample | TΔM (g/mol) | AΔM (g/mol) | TΔM/AΔM |
| --- | --- | --- | --- | --- |
| Mn | BAM-HA | 9,640.70 | 43,724 | 22.05% |
|  | BAM-LA | 9,640.70 | 65,941 | 14.62% |
|  | BAM-AA | 9,640.70 | 81,546 | 11.82% |
| Mw | BAM-HA | 23,859.60 | 80,258 | 29.73% |
|  | BAM-LA | 23,859.60 | 121,987 | 19.56% |
|  | BAM-AA | 23,859.60 | 203,198 | 11.74% |
| Mp | BAM-HA | 17,842.60 | 30,669 | 58.18% |
|  | BAM-LA | 17,842.60 | 44,275 | 40.30% |
|  | BAM-AA | 17,842.60 | 69,314 | 25.74% |

Through an in-depth cross-analysis of the four tables (Table S4 and Table S5) based on the "actual lipid content (LC)" and the "10% feed ratio" for both relatively high and low molecular weight (BAM and AAM) systems, it is evident that the dramatic increase in the apparent molecular weight of amylose-lipid complexes RS5) is by no means a simple physical mass addition of the guest molecules.

First, based on calculations using the actual lipid content, the theoretical contribution rate of the physical mass is extremely low, regardless of whether short-chain or long-chain fatty acids are complexed. For the short-chain hexanoic acid (HA), the theoretical increment in the number-average molecular weight (TMn) accounts for only 4.04% and 2.68% of the actual GPC increments for BAM and AAM, respectively. As the carbon chain lengthens to lauric acid (LA) and arachidic acid (AA), although the lipid loading increases significantly, the physical mass increment remains minimal. In the long-chain AA system, the theoretical ΔMn (TΔM) increments (6,903.2 g/mol for BAM and 799.5 g/mol for AAM) can only explain 8.47% and 9.86% of their massive actual increments (AΔM: 81,546 g/mol and 8,107 g/mol), respectively.

Second, upon introducing the 10% limit feed ratio model (assuming 100% perfect complexation of the 0.3 g fatty acids), the maximum theoretical physical mass increments for the HA, LA, and AA systems are completely fixed (9,640.7 g/mol for the BAM system and 1,551.4 g/mol for the AAM system). However, against the backdrop of this strictly constrained absolute physical increment, the actual apparent molecular weight exhibits an astonishing step-wise escalation with increasing chain length (HA < LA < AA). Under this limit model, the proportion of the actual increment (AΔM) explained by the theoretical limit increment (TΔM) drops sharply from 22.05% (BAM) and 22.81% (AAM) in the HA system down to 11.82% and 19.14% in the AA system.

Therefore, a deduction we propose is that the carbon chain length of the fatty acid might act as a switch. Although the long-chain arachidic acid (AA) possesses the lowest molar amount under the equivalent feed mass, it relies on its exceptionally strong intermolecular hydrophobic interactions (with a binding energy reaching -238.74 kJ/mol) to function as a powerful physical cross-linking agent, inducing severe conformational changes within the amylose network. Compared to the loose, porous structures formed by HA, AA drives the starch to aggregate highly. The aforementioned phenomena by no means imply that the mass of the fatty acids is unimportant. In fact, the actual mass of the incorporated fatty acids (i.e., the experimentally determined lipid content, LC) plays an equally crucial role in the fine structural assembly of the RS5 complexes. Consequently, the above phenomena and deductions still require further verification.

**Table S6 Particle size data of the sample.**

| Sample | Specific surface area (m²/kg) | D [3,2] (μm) | D [4,3] (μm) | Dx (10) (μm) | Dx (50) (μm) |
| --- | --- | --- | --- | --- | --- |
| AAM | 546.80 ± 13.96^d^ | 10.97 ± 0.28^a^ | 23.90 ± 0.42^a^ | 7.77 ± 0.20^a^ | 20.17 ± 0.74^a^ |
| AAM-HA | 775.90 ± 5.49^c^ | 7.73 ± 0.06^b^ | 11.91 ± 0.81^c^ | 3.85 ± 0.06^b^ | 10.79 ± 0.05^b^ |
| AAM-LA | 839.00 ± 9.34^b^ | 7.15 ± 0.08^c^ | 13.78 ± 0.73^b^ | 3.52 ± 0.09^c^ | 9.92 ± 0.03^b^ |
| AAM-AA | 972.10 ± 17.93^a^ | 6.17 ± 0.12^d^ | 11.08 ± 0.40^c^ | 3.01 ± 0.05^d^ | 8.39 ± 0.19^c^ |
| BAM | 78.12 ± 2.80^d^ | 76.80 ± 2.79^a^ | 462.74 ± 47.48^a^ | 30.85 ± 1.19^a^ | 278.69 ± 20.00^a^ |
| BAM-HA | 112.10 ± 2.77^c^ | 53.53 ± 1.37^b^ | 380.41 ± 27.57^ab^ | 22.33 ± 0.63^b^ | 178.91 ± 10.15^b^ |
| BAM-LA | 175.90 ± 6.07^b^ | 34.11 ± 1.21^c^ | 281.04 ± 57.45^b^ | 14.08 ± 0.36^c^ | 78.76 ± 8.41^d^ |
| BAM-AA | 197.00 ± 7.82^a^ | 30.46 ± 1.20^c^ | 271.24 ± 39.36^b^ | 10.20 ± 0.44^d^ | 130.09 ± 8.42^c^ |

Significance analysis of Columns a, b, c, d, e, and f. D [4, 3]: Volume weighted mean; D [3,2]: Surface weighted mean; Dx (10), Dx (50), and Dx (90) represent the diameter of particles with a volume fraction integral of 10 %, 50 %, and 90 %, respectively.
